# Characteristics of HPV-Specific Antibody Responses Induced by Infection and Vaccination: Cross-Reactivity, Neutralizing Activity, Avidity and IgG Subclasses

**DOI:** 10.1371/journal.pone.0074797

**Published:** 2013-09-18

**Authors:** Mirte Scherpenisse, Rutger M. Schepp, Madelief Mollers, Chris J. L. M. Meijer, Guy A. M. Berbers, Fiona R. M. van der Klis

**Affiliations:** 1 Laboratory for Infectious Diseases and Screening, National Institute of Public Health and the Environment, Bilthoven, The Netherlands; 2 Department of Epidemiology and Surveillance, National Institute of Public Health and the Environment, Bilthoven, The Netherlands; 3 Department of Pathology, VU University Medical Center, Amsterdam, The Netherlands; University of Cape Town, South Africa

## Abstract

**Objectives:**

In order to assess HPV-specific IgG characteristics, we evaluated multiple aspects of the humoral antibody response that will provide insight in the HPV humoral immune response induced by HPV infection and vaccination.

**Methods:**

Cross-reactivity of HPV-specific antibodies induced by infection or vaccination was assessed with VLP16 or 18 inhibition using a VLP-based multiplex immunoassay (MIA) for HPV16, 18, 31, 33, 45, 52 and 58. HPV16/18 specific IgG1-4 subclasses and avidity were determined with the VLP-MIA in sera after HPV infection and after vaccination. Neutralizing antibodies were determined in a small subset of single-seropositive and multi-seropositive naturally derived antibodies.

**Results:**

Naturally derived antibodies from single-positive sera were highly genotype-specific as homologue VLP-inhibition percentages varied between 78-94%. In multi-positive sera, cross-reactive antibodies were observed both within and between α7 and α9 species. After vaccination, cross-reactive antibodies were mainly species-specific. Avidity of vaccine-derived HPV-specific antibodies was 3 times higher than that of antibodies induced by HPV infection (*p*<0.0001). IgG1 and IgG3 were found to be the predominant subclasses observed after HPV infection and vaccination. In the small subset tested, the number of single-positive sera with neutralizing capacity was higher than of multi-positive sera.

**Conclusion:**

Naturally derived HPV-specific antibodies from single-positive samples showed different characteristics in terms of cross-reactivity and neutralizing capacity compared with antibodies from multi-positive sera. Post-vaccination, HPV antibody avidity was approximately 3 times higher than antibody avidity induced by HPV infection. Therefore, antibody avidity might be a potential surrogate of protection.

## Introduction

Persistent infection with high-risk (hr) human papillomavirus (HPV) is a necessary event of cervical cancer. Almost all cervical cancers are HPV DNA-positive to at least one of the 15 hr-HPV types that can cause genital infections. Most hr-HPV types cluster together in the α7 and α9 species [1,2].

After an incidental HPV infection, only 50-70% of the infected individuals develop detectable HPV-specific antibodies in serum mainly directed against the L1 capsid protein of the virus [3]. In contrast to the weak immune response induced by naturally HPV infections, the HPV vaccines induce strong immunogenic responses against HPV16 and 18 in serum [4]. The HPV-specific antibody levels after vaccination in serum are 10-100 times higher as compared to naturally derived antibody levels. Vaccine-derived antibody levels are thought to be largely responsible for the protection against subsequent infection and cervical intraepithelial neoplasia (CIN), the precursors of cervical cancer [4,5].

HPV vaccines provide a degree of cross-protection against persistent infection and/or high-grade lesions (CIN2+) attributed to some of these non-vaccine HPV genotypes, particularly HPV31, 33 and 45, but probably not HPV52 and 58 [6,7]. It is not clear whether naturally derived HPV-specific antibodies can also protect against subsequent homologous infection or against infection with phylogenetically related HPV genotypes [8,9].

At present, no immune parameter or antibody concentration has been defined that correlates with protection. While the HPV-specific antibody levels in response to HPV infection or HPV vaccination have been previously described [10-13], information about HPV-specific antibody avidity or IgG subclasses is scarce [14-16]. In this study, we evaluate multiple aspects of the humoral antibody response induced by HPV infection and after prophylactic HPV vaccination. Therefore, we determined, next to the antibody concentration, the antibody avidity, IgG subclasses, cross-reactive antibodies and neutralizing activity of antibodies induced by HPV infection for HPV genotypes of the α7 (HPV18 and 45) and α9 species (HPV16, 31, 33, 52 and 58). These antibody characteristics will provide insight in the HPV humoral immune response, which is important for the interpretation of sero-epidemiological data and might contribute to a definition of an HPV-specific correlate of protection.

## Methods

### Study populations

#### Naturally infected individuals

Serum samples were available from a large cross-sectional population-based surveillance study (PIENTER-2), performed in 2006-07 in the Netherlands. This serumbank contained samples from individuals 0-79 years of age and at low risk for HPV infection [17]. The surveillance study design and the study proposal were approved by the Medical Ethics Testing Committee of the foundation of therapeutic evaluation of medicines (METC-STEG) in Almere, the Netherlands (clinical trial number: ISRCTN 20164309). A signed informed consent was obtained from all participants and for those below 18 years of age also from the parents, care takers or guardians.

From this serosurveillance study, a panel of naturally HPV infected individuals was selected that included HPV single-positive (*n*=121) and multi-positive sera (at least seropositive for HPV16 (*n*=136) or seropositive for HPV18 (*n*=91)) of a broad range of antibody concentrations [12]. The vast majority of the individuals included in this selection were older than 15 years of age.

Some infants and children (up to 10 years of age) in the large serosurveillance study were HPV antibody seropositive. To investigate the quality of the antibodies we tested in the PBNA some sera of infants and children, who were part of the previous mentioned selection (n=11).

From a prospective cohort study in Rwanda, Africa [18], a panel of sera from women, who were at high risk for HPV infection, was selected for IgG subclass determination because of the high HPV antibody concentrations in these sera.

#### Vaccinated individuals

From an HPV vaccine monitoring study (HAVANA study, HPV amongst vaccinated and non-vaccinated adolescents [19]) in the Netherlands, serum samples of girls aged 14-16 years, 12 months after the first vaccine dose were available (*n*=100). The girls from the HAVANA study received three doses of the bivalent Cervarix^®^ vaccine (GlaxoSmithKline, Rixensart, Belgium) at months 0, 1 and 6. The study protocol was approved by a medical ethics review committee of the Faculty of Medicine, VU University Amsterdam (2009/22).

### Serological measurements

#### VLP-based multiplex immunoassay

HPV-specific IgG antibodies against L1 VLP16, 18, 31, 33, 45, 52 and 58 were measured using a VLP-MIA [12]. Sera were incubated with the VLP-coupled microspheres. HPV-specific IgG antibodies were detected using R-phycoerythrin (R-PE) conjugated goat anti-human IgG (Jackson ImmunoResearch laboratories Inc, Westgrove, PA). For the determination of HPV16/18-specific IgG subclasses (IgG1, IgG2, IgG3 and IgG4) after HPV infection (HPV16 *n*=67, HPV18 *n*=29) and vaccination (HPV16 *n*=64, HPV18 *n*=63), isotype-specific mouse anti-human R-PE-conjugated antibodies were used. The mouse anti-human IgG1 monoclonal was used in a 1/500 dilution and the IgG2-4 monoclonals in a 1/100 dilution (all 4 from SouthernBiotech, Birmingham, AL). Four ‘in-house’ control sera and an ‘in-house’ standard were used on each plate. The ‘in-house’ standard (IVIG, lot LE12H227AF, Baxter) was calibrated against reference serum of GSK for all the seven HPV types. HPV-specific antibodies were analyzed using the Bioplex system 200 with Bioplex software (Bio-Rad Laboratories, Hercules, CA). Sera were assumed to be IgG seropositive at the following cut-offs determined previously with this assay [12]: 9, 13, 27, 11, 19, 14 and 31 LU/ml for HPV16, 18, 31, 33, 45, 52 and 58, respectively. Distributions of IgG subclasses in percentages were calculated using median fluorescent intensity (MFI) of the IgG subclasses separately in relation to the MFI of HPV-specific total IgG, which was set at 100%.

#### VLP16 and 18-based inhibition assay

Homologous inhibition of HPV-specific antibodies induced by infection was assessed for single-positive sera with virus-like particle (VLP) 16, 18, 31, 33, 45, 52, and 58, separately. Inhibition of HPV antibodies in multi-positive sera from the Dutch serosurveillance study and sera from vaccinated girls was determined with VLP16 and 18 separately. VLPs were kindly donated by GlaxoSmithKline Vaccines. Sera were pre-diluted 1/100 and 1/200, respectively, and subsequently added to the same volume of PBS containing 10 µg/ml VLPs. Sera, containing vaccine-derived HPV-specific antibodies, were pre-diluted 1/10,000. After an incubation period at RT for 1 hour, all samples were analyzed using the VLP-based multiplex immunoassay (VLP-MIA). The inhibition index was determined as a percentage of the antibody levels post-inhibition in comparison with the antibody levels pre-inhibition, which were set at 100%.

#### Antibody avidity measurement

Avidity of HPV16 and 18-specific IgG antibodies was assessed by using a modification of the VLP-MIA for determining HPV-specific IgG antibodies. HPV16 and 18-specific antibody concentrations in sera induced by HPV infection (HPV16 *n*=65, HPV18 *n*=55) or after HPV vaccination (HPV16/18 *n*=60) were diluted for HPV16 and 18 separately and adapted to a starting concentration of 0.25 LU/ml (approximately 6000 MFI, which is the middle of the linear part of the reference dilution curve).

Ammonium thiocyanate (NH_4_SCN; Sigma-Aldrich, St. Louis, Missouri, USA) was used to dissociate low-avidity antigen-antibody binding [20]. After a 60 minute incubation period of VLP-coupled microspheres with serum, 25 µl of NH_4_SCN in PBS (pH7.2) was added in a final concentration of 2.5M or only PBS for 10 minutes at RT. Subsequently, sera were washed 3 times with PBS. Residual bound IgG antibodies were detected as described above. The avidity index was determined as a percentage of the remaining IgG levels in the presence of NH_4_SCN in comparison with the IgG levels after addition of PBS in which the antibody avidity was set at 100%.

#### Pseudovirion-based neutralization assay

Subsets of single-positive (*n*=11) and multi-positive (*n*=10) sera, containing naturally induced HPV16, 18, 31 and 45-specific antibodies, were tested in a pseudovirion-based neutralization assay (PBNA) for the detection of neutralizing antibodies against VLP16, 18, 31 and 45. The PBNA was performed at GSK as previously described [21]. Serum neutralization titers were calculated by linear interpolation of the serum dilutions of every sample and defined as the reciprocal of the serum dilution, which caused 50% reduction in secreted alkaline phosphatase activity (ED_50_, when compared to control wells containing only neutralization buffer and HPV pseudovirions). The cut-off of the PBNA was 40 ED_50_ for HPV16, 18, 31 and 45.

### Statistical analysis

Data analyses were conducted using Graphpad Prism version 5. Percentage inhibition was calculated using the antibody concentrations pre- and post-inhibition of each tested sample. Subsequently, an average inhibition percentage was calculated. Significant differences (*p*<0.05) of geometric mean concentrations (GMCs) of antibodies pre- and post-inhibition or between antibody avidity indices were calculated using a Mann-Whitney test.

**Figure 1:**
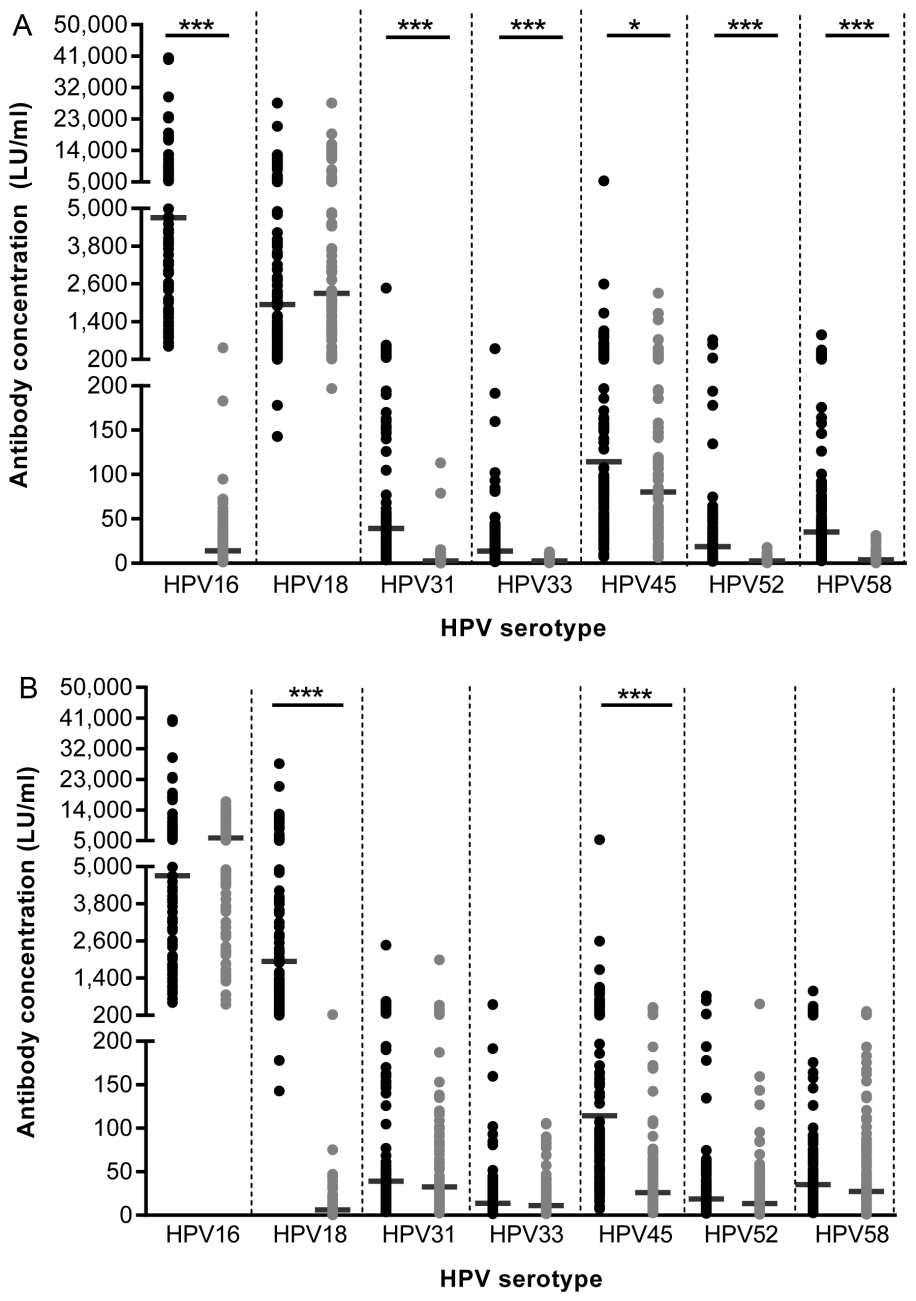
Inhibition of vaccine-derived HPV16 and 18 antibodies. Vaccine-derived antibody concentrations (LU/ml) pre- (dark grey dots) and post-inhibition (light grey dots) with VLP16 (A) or VLP18 (B) are shown. Dark grey lines indicate geometric mean concentrations. * *p*=0.05, *** *p*<0.0001.

## Results

### Cross-reactivity of HPV-specific antibodies derived after HPV vaccination

Homologous inhibition of vaccine-derived HPV16 and 18-specific antibodies was nearly complete and amounted to 99.7% and 99.6%, respectively (Figure 1A and 1B). Therefore, vaccine-derived HPV-specific antibodies were genotype specific.

After vaccination, cross-reactive antibody levels were mainly species-specific. A significant reduction in antibody levels of phylogenetically related HPV types was observed after VLP16 inhibition with inhibition percentages varying between 76% and 88% for HPV31, 33, 52 and 58 (Figure 1A). However, we also found a small decline in HPV45 antibody levels (28%). The reduction in antibody levels after VLP18 inhibition was also mainly species-specific and the inhibition percentage for the α9 species (HPV45) was 73% (Figure 1B). Small reductions in antibody levels varying between 18% and 27% were observed after VLP18 inhibition for the α7 species (HPV31, 33, 52 and 58).

**Figure 2:**
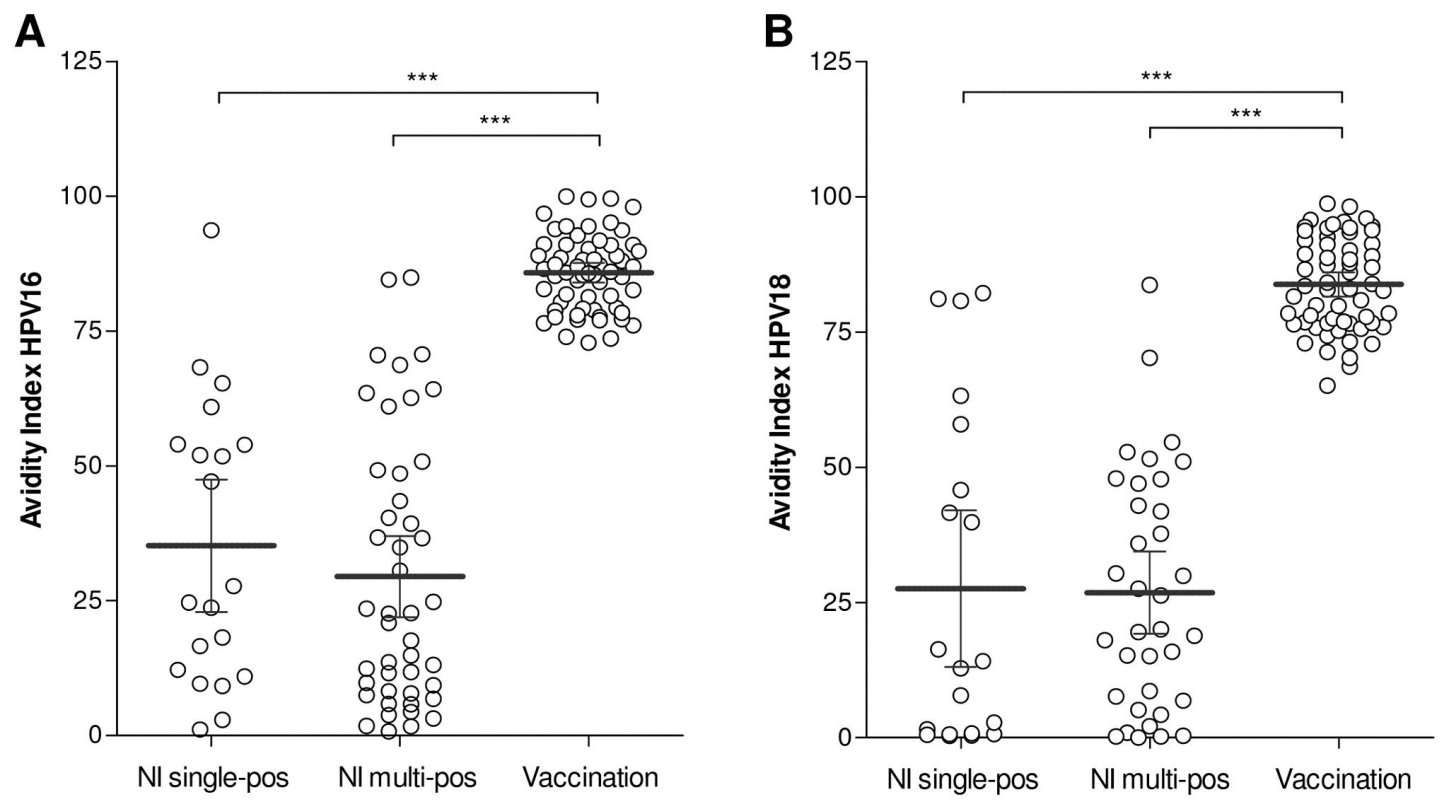
Antibody avidity after HPV infection and vaccination. Antibody avidity (%) for HPV16 (A) and HPV18 (B) of HPV-specific single-seropositive and multi-seropositive naturally derived antibodies and HPV vaccine-derived antibodies are shown. The dark grey line indicates the mean antibody avidity. *** *p*<0.0001.

**Table 1:** VLP16 and 18 homologous inhibition and heterologous inhibition percentages of multi-positive sera induced by HPV infection.

	**Inhibition with VLP16**			**Inhibition with VLP18**		
	**Homologous inhi (%**)	**Heterologous inhi (%**)		**Homologous inhi (%**)	**Heterologous inhi (%**)	
**Types positive**	**HPV16**	**α9 - HPV16**	**α7**	**HPV18**	**α9**	**α7 - HPV18**
2 types	78	42	39	83	76	38
3 types	80	23	44	72	62	55
4 types	75	54	56	85	61	65
5 types	69	47	44	78	48	43
6 types	71	56	57	81	65	56
2-6 types	75	45	48	80	62	51

The percentage of VLP16 an 18 inhibition within and between the α7 and α9 species are shown for sera antibody seropositive for 2 up to 6 HPV types.

Note: inhi = inhibition, α9: HPV16, 31, 33, 52, 58, α7: HPV18, 45.

### Cross-reactivity of HPV-specific antibodies induced by HPV infection

Homologous inhibition of naturally acquired HPV-specific antibodies against a single HPV type amounted to 94.5% for HPV16 and 83.1% for HPV18 (*p*<0.0001 for both HPV16 and HPV18). Homologous inhibition percentages for the other 5 single-seropositive HPV types tested varied between 78% and 92%, indicating that these antibodies were type-specific as expected (data not shown).

In contrast to vaccine-derived and naturally induced antibodies of single-positive sera, antibodies of multi-positive sera were less specific. In sera positive for HPV16 or HPV18 and up to 5 other HPV types, inhibition with VLP16 or 18 amounted to an average percentage of 75% and 80%, respectively (Table 1). Heterologous inhibition with VLP16 or VLP18 of these sera multi-positive for 2-6 HPV types reduced the antibody levels of phylogenetically related HPV types both within and between species α7 and α9 varying between 45 and 62%.

### HPV-specific neutralizing antibodies induced by HPV infection in single-positive and multi-positive sera

A subset of sera with high homologous inhibition percentages (98% for HPV16 and 97% for HPV18), indicating that the antibodies were genotype-specific, was selected from the single- and multi-positive samples. In the single-positive sera from this subset, neutralizing antibodies were found in 5 out of 6 for HPV16 and in 4 out of 5 for HPV18 (Table 2). In the multi-positive sera from this subset, the naturally derived HPV-specific antibodies showed less neutralizing activity than HPV-specific antibodies of single-positive sera (Table 3). Although these samples were selected for high HPV16 and 18 inhibition percentages, only 1 out of 6 for HPV16 and 1 out of 4 for HPV18 showed neutralizing capacity. Antibodies that cross-reacted with HPV31 and HPV45 showed no neutralizing capacities.

**Table 2:** HPV16 and 18 antibody characteristics of single-positive sera induced by HPV infection.

		**VLP-MIA**		**PBNA**	**Avidity**
	**#**	**Conc (LU/ml**)	**Inhi (%**)	**ED_50_**	**%**
**HPV16**	1	138	99	591	54
	2	105	98	297	54
	3	105	99	110	52
	4	99	100	<40	10
	5	92	97	263	25
	6	41	97	100	65
**HPV18**	7	130	99	94	81
	8	96	97	108	3
	9	57	99	187	81
	10	49	94	<40	1
	11	28	94	62	16

Antibody concentrations (LU/ml) assessed with the VLP-MIA, homologous inhibition percentages, neutralizing antibody levels (ED_50_) and antibody avidity indices are presented for HPV16 and HPV18.

Note: Conc, concentration, Inhi, inhibition, PBNA antibody levels <40 were not neutralizing

**Table 3:** HPV-specific antibody characteristics of multi-positive sera induced by HPV infection.

		**VLP-MIA**				**PBNA**		**Avidity**
	**#**	**Conc (LU/ml**)		**Inhi (%**)		**ED_50_**		**%**
		**16**	**31**	**16**	**31**	**16**	**31**	**16**
**HPV16+31**	1	224	131	99	74	<40	<40	63
	2	142	42	98	98	<40	<40	4
	3	27	51	85	77	<40	<40	1
		**16**	**45**	**16**	**45**	**16**	**45**	**16**
**HPV16+45**	4	27	28	97	68	<40	<40	37
	5	19	26	94	74	127	<40	12
	6	9	29	79	69	<40	<40	24
		**18**	**45**	**18**	**45**	**18**	**45**	**18**
**HPV18+45**	7	121	52	99	96	209	<40	1
	8	40	162	97	94	<40	<40	53
	9	37	35	97	76	<40	<40	30
	10	25	20	96	61	<40	<40	15

Antibody concentrations (LU/ml) assessed with the VLP-MIA, homologous and heterologous inhibition percentages and neutralizing antibody levels (ED_50_) are shown for HPV16, 18, 31 and 45. Antibody avidity indices are presented for HPV16 and 18.

Note: Conc, concentration, Inhi, inhibition, PBNA antibody levels <40 were not neutralizing

Naturally derived HPV16-specific antibodies in serum samples of children 1-10 years of age (n=8) showed no neutralizing capacity while from the samples of 3 infants tested (0-6 months of age) in 1 case HPV-specific neutralizing antibodies were found.

### HPV16 and 18-specific antibody avidity induced by HPV infection and vaccination

HPV16 and 18-specific antibodies induced by HPV infections are mainly of low-avidity (Figure 2A and 2B). On average, 35% (95%CI 23-47%) and 28% (95%CI 13-42%) of HPV16 and 18-specific antibodies of single-positive sera, respectively, remained detectable after NH_4_SCN treatment. No significant differences in naturally induced antibody avidity were observed between single-positive and multi-positive sera. In contrast, vaccine-derived HPV16 and 18 antibodies showed a significant 3 times higher avidity than naturally induced antibodies, with an avidity index of 86% (95%CI 84-88%) and 84% (95%CI 82-86%), respectively. The avidity indices of the antibodies induced by HPV infection of the vast majority of the single-positive (19/20 for HPV16, 17/20 for HPV18) and multi-positive sera (43/45 for HPV16, 33/35 for HPV18) were lower than the avidity of vaccine-derived antibodies. We observed no correlation between the level of antibody avidity and the number of HPV types for which participants are positive. Also no correlation was observed between antibody avidity indices and the level of HPV16 and 18-specific antibody concentrations after HPV infection and vaccination (data not shown).

### HPV16 and 18-specific IgG subclass antibody distributions after HPV infection and vaccination

The most abundant antibody subclass of IgG induced after HPV16 and 18 infection was IgG1 (78% and 92%, respectively), followed by IgG3 (20% and 6%, respectively) (Table 4). Small amounts of IgG2 and IgG4 subclass were found, 2% and 1% for HPV16 and 1% and 2% for HPV18, respectively. Similar IgG isotype distributions were observed after HPV vaccination, although IgG2 and IgG4 tended to be even lower.

**Table 4:** HPV16 and HPV18 IgG subclasses (IgG1, IgG2, IgG3 and IgG4) induced by HPV infection (HPV16 n=67, HPV18 n=29) and vaccination (HPV16 n=64, HPV18 n=63).

	**Natural Infection**					**Vaccination**			
	**HPV16**		**HPV18**			**HPV16**		**HPV18**	
	%	95%CI	%	95%CI		%	95%CI	%	95%CI
IgG1	78	71-84	92	86-98		80	73-87	70	61-78
IgG2	2	1-4	1	0-1		0	0-1	1	0-3
IgG3	20	13-26	6	0-11		19	13-26	26	19-34
IgG4	1	0-1	2	0-6		0	0-1	3	1-4

Proportions of IgG isotypes are expressed as percentages towards total HPV-specific IgG.

Note: CI, Confidence interval

## Discussion

Here we describe HPV-specific antibody characteristics after HPV infection and vaccination. Cross-reactivity of HPV antibodies derived after HPV vaccination was mainly species-specific. Naturally induced HPV-specific antibodies from single-positive sera were genotype-specific as expected and tended to be neutralizing. In contrast, antibodies of multi-positive sera were less genotype-specific, cross-reactive, and tended to be non-neutralizing. IgG1 was found the predominant subclass induced after HPV infection and vaccination, followed by IgG3. Moreover, post-vaccination antibody avidity was approximately 3 times higher than after HPV infection.

### Vaccine-derived HPV16 and 18-specific and cross-reactive antibodies

Vaccine efficacy against HPV16 and 18 infection sustains over 8 years post-vaccination [4] in which HPV-specific IgG antibody levels together with its neutralizing activity remained well above antibody levels induced by HPV infection [4,16]. In addition, HPV vaccines offer cross-protection against a few non-vaccine HPV types in individuals without a previous HPV infection [22]. Antibodies that were capable of neutralizing also non-vaccine HPV types were most frequently found to be directed against HPV31 and 45 [23]. Cross-neutralizing antibody levels against HPV31, 33, 35 and 45 were found to be significantly associated with their phylogenetically related vaccine type antibody levels [23]. We showed that 12 months after the first vaccine dose, vaccine-derived HPV16 and 18 antibody levels were indeed cross-reactive as inhibition with VLP16 or 18 resulted in species-specific inhibition of antibodies directed against HPV31, 33, 45, 52 and 58. The HPV types 31, 33, and 45 are often detected in cervical cancer worldwide and vaccines that are efficacious against these HPV types might further reduce cancer incidence. However, vaccine efficacy against non-vaccine HPV types decreased rapidly over time [22]. A short duration of cross-protection could limit the reduction in cancer against cross-reactive HPV types. Although we found vaccine-derived HPV52 and 58 cross-reactive antibody levels to be associated with HPV16, these cross-reactive antibodies are probably of limited biological relevance. Vaccine efficacy studies did not observe cross-protection against HPV52 and 58 as these HPV types are more distantly related to HPV16 [7].

### Naturally derived cross-reactive antibodies

It is unclear whether naturally derived HPV-specific antibodies can protect against subsequent homologous HPV infection or infection with phylogenetically related HPV types. In women, the protective role of HPV16 serum antibodies induced by HPV infection has been inconsistent in the literature, with moderate protection observed in a limited number of studies [8,24-26]. Palmroth et al. did not find cross-protection with naturally induced HPV16 or HPV18 antibodies against infection with phylogenetically related genotypes of α7 or α9 species among single-seropositive women [9]. We also found that naturally induced HPV16 and 18 antibodies in single-positive sera were non-cross-reactive. In addition, we found that single-positive sera showed neutralizing capacity while antibodies in multi-positive sera were cross-reactive and non-neutralizing, albeit tested in a small subset. We hypothesize that in single-positive sera the HPV-specific antibodies might be induced by transient HPV infections, whereas for multi-positive sera the HPV-specific antibodies might be induced after persistent HPV infections. A high viral load could induce an acute and strong immune response [27,28]. This might result in an effective HPV type-specific humoral immune response, in which it is more likely that naturally induced antibodies are directed against HPV-specific neutralizing epitopes. Cross-reactive HPV antibodies in multi-HPV type positive sera might be induced by, i.e. persistent HPV infections that generate and maintain a less developed humoral immune response with a slow gradual boosting effect of the HPV antibodies. Moreover, continued exposure to more than one HPV genotype may favour the induction of HPV-specific antibodies that are directed against the common non-neutralizing epitopes shared by different HPV genotypes. This might denote that naturally induced HPV-specific antibodies in multi-HPV positive individuals could not or only partially protect against subsequent HPV infection and that HPV seropositive individuals are still at risk for subsequent infection with related HPV types. Of course we realise that these explanations are speculative and need further immunological corroboration. The low inhibition percentages of naturally induced antibodies in multi-positive sera might be due to the presence of antibodies directed against epitopes that are specific for the non-HPV16 and 18 HPV types. Although these HPV types are phylogenetically related to HPV16 and 18, VLP16 and 18 could not inhibit these HPV type specific antibodies. In addition, cross-reactive HPV antibodies (recognizing common epitopes) not measured in the VLP-MIA, of the α7 and α9 species, could also contribute to the low inhibition percentages. Cross-reactive antibodies induced by HPV infection, might play an important role in the interpretation of HPV sero-epidemiological data from cross-sectional studies. Although cross-reactivity after HPV infection is relatively rare [12], it can lead to an overestimation of seropositivity and indirectly to an overestimation of HPV infection rates.

Cross-sectional serosurveillance studies [12,29] showed that naturally induced HPV-specific antibodies could be detected in sera of infants and children (0-10 years of age). However, whether these HPV-specific antibodies have any biological relevance in terms of neutralizing capacity in children and infants has not been investigated. We showed, in a small subset, that naturally derived HPV-specific antibodies in children were non-neutralizing. HPV seropositivity in children might be associated with non-sexual transmission of HPV, as HPV-DNA can be detected in the oral mucosa, tonsils and fingers [29-31]. However, HPV-specific antibodies in children might also be cross-reactive antibodies against other HPV types e.g. cutaneous HPV types which are highly prevalent in children. The neutralizing HPV-specific antibodies in 1 infant (age 3 months) can be explained by transfer of maternal antibodies that subsequently wane within a few months after birth [29,32].

### Potential surrogates of protection

With the HPV vaccine clinical trials performed so far, a level of HPV-specific antibodies that can be used as a correlate of protection is still not established as too few disease cases occurred within these trials and breakthrough infections could not be clearly distinguished from the reactivation of prevalent HPV infections [33]. Longet et al. showed in mice, by *in vivo* genital HPV pseudovirion challenge, that already very low vaccine-derived HPV-specific antibody levels could protect against HPV infections and that these protective antibody levels are far below detection limits of the pseudovirion-based neutralization assay [33]. We could not find an association between the level and the neutralizing capacity of naturally induced antibodies. This is in line with a study of Lu et al., showing that the level of HPV16 antibodies was not associated with a lower risk of HPV infection [34]. Apparently, the HPV antibody level after infection will provide limited information about a correlate of protection.

Other immune parameters than antibody levels, that might correlate with protection, have not been defined and data on antibody characteristics such as avidity are scarce [16]. Antibody avidity generally increases over time following encounter with an antigen. Memory responses are characterized by the production of high-avidity antibodies. Thus, the level of antibody avidity could be considered a surrogate of successful induction of immunological memory [35]. Vaccine-derived neutralizing antibody levels correlate with antibody avidity 6 months and one year after HPV vaccination [15,16].

We found that antibody avidity after vaccination was 3 times higher than after HPV infection. After HPV infection, we observed a wide range of antibody avidity levels in sera of naturally infected individuals reflecting a great biological diversity in individual responses to HPV infection. Naturally induced HPV16 antibodies of low avidity have been associated with possible susceptibility to infection with other HPV types [14]. Higher avidity indices tended to be associated with naturally derived neutralizing HPV-specific antibodies, albeit tested in small sample sizes. This indicates that levels of antibody avidity might be useful to distinguish between protective and non-protective HPV-specific antibodies. Therefore, antibody avidity might be a potential immune surrogate of HPV protection, but further research is necessary to confirm this.

Our findings on IgG subclasses are in line with Harro et al. reporting that IgG1 was the predominant subclass induced after VLP vaccination [36]. In contrast, Matsumoto et al. found the IgG2 subclass to be dominant in HPV16 seropositive women and that IgG2 dominance was associated with the regression of CIN, albeit in a small sample size [37]. IgG1 and IgG3 are generally induced in response to protein antigens, whereas IgG2 is associated with the immune response against polysaccharide antigens and IgG4 with allergy [38,39].

In conclusion, we showed that vaccine-derived antibodies were mainly genotype-specific and cross-reacted only for a smaller part with other HPV types within the species. After HPV infection, single-seropositive antibodies were highly type-specific and neutralizing whereas multi-positive sera were less specific and tended to be non-neutralizing. This might mean that naturally induced HPV-specific antibodies in multi-HPV positive individuals could not or only partially protect against subsequent HPV infection and these individuals are still at risk.

Although sample sizes were small, neutralizing antibodies in single-positive sera inclined to be associated with higher antibody avidity indices than non-neutralizing antibodies. Further, we found the avidity of vaccine-derived antibodies to be approximately 3 times higher than after HPV infection. These results imply that the avidity of HPV antibodies might be used as a potential surrogate of protection. However, more studies are needed to establish the role of HPV antibody avidity as a potential surrogate of protection and the use of this immunological tool in sero-epidemiological and vaccine monitoring studies.
